# Clinical Utility of GnRH Analogues in Female Androgen Excess: Highlighting Diagnostic and Therapeutic Applications

**DOI:** 10.1210/jcemcr/luad108

**Published:** 2023-09-19

**Authors:** Lauren Madden Doyle, Leanne Cussen, Tara McDonnell, Michael W O'Reilly

**Affiliations:** Academic Division of Endocrinology, Department of Medicine, Royal College of Surgeons in Ireland, Dublin 9, Ireland; Department of Endocrinology, Beaumont Hospital, Dublin 9, Ireland; Academic Division of Endocrinology, Department of Medicine, Royal College of Surgeons in Ireland, Dublin 9, Ireland; Department of Endocrinology, Beaumont Hospital, Dublin 9, Ireland; Academic Division of Endocrinology, Department of Medicine, Royal College of Surgeons in Ireland, Dublin 9, Ireland; Department of Endocrinology, Beaumont Hospital, Dublin 9, Ireland; Academic Division of Endocrinology, Department of Medicine, Royal College of Surgeons in Ireland, Dublin 9, Ireland; Department of Endocrinology, Beaumont Hospital, Dublin 9, Ireland

**Keywords:** androgen excess, GnRH analogue suppression, virilizing ovarian tumor, severe insulin resistance syndrome

## Abstract

Female androgen excess typically presents with hirsutism, acne, and frontotemporal alopecia. Although the majority of cases are due to underlying polycystic ovary syndrome, non-polycystic ovary syndrome pathology can present a diagnostic and therapeutic challenge. We present 3 cases highlighting the utility of GnRH analogues in diagnosis and treatment of ovarian hyperandrogenism. In case 1, we highlight the role of GnRH analogue testing to localize severe postmenopausal androgen excess, allowing full resolution of symptoms following resection of a benign ovarian steroid-cell tumor. Our second case demonstrates the dual utility of GnRH analogues as both a diagnostic and therapeutic agent for hyperandrogenism in a premenopausal woman with severe insulin resistance. We observed suppression of serum testosterone coupled with significant improvement in hirsutism scores. The final case describes GnRH analogue suppression as a therapeutic option for a postmenopausal woman with ovarian hyperthecosis wishing to avoid surgical intervention, with successful symptom resolution. This case series delineates the applications of GnRH analogue suppression in a variety of clinical contexts, in particular their potential role in controlling symptoms in cases of refractory androgen excess and an alternative to surgery in cases of benign ovarian hyperandrogenism.

## Introduction

Clinical features of androgen excess in women occur from increased circulating androgenic steroids such as testosterone or its precursors, which may be of ovarian or adrenal origin. Polycystic ovary syndrome (PCOS) is the most common cause of hyperandrogenism in women and typically presents with mild to moderate symptoms including hirsutism, frontotemporal alopecia, and acne [1]. Non-PCOS pathology should be considered in the context of atypical features including rapid symptom onset, overt virilization, or severe biochemical abnormalities [2].

Rare causes of androgen excess represent a diagnostic conundrum, with further challenges posed by their presence alongside patients with PCOS in busy endocrinology clinics. The lack of sensitivity and specificity of current imaging approaches for ovarian neoplastic pathology means that other diagnostic approaches should be considered. GnRH analogues can be used diagnostically to confirm the presence of underlying gonadotropin-dependent ovarian disease and therapeutically in patients in which surgical intervention is deemed high risk or undesirable by the patient [3, 4].

In this case series, we present 3 cases highlighting the clinical application of GnRH analogues and propose a role for further expansion in diagnostic and therapeutic algorithms for managing hyperandrogenism.

## Case Presentation

### Case 1

A 68-year-old woman presented with a 3- to 4-year history of hirsutism, requiring frequent topical cosmetic interventions. Additional symptoms included frontotemporal alopecia, altered body odor, and deepening of the voice, and changes in size and appearance of the external genitalia. Her medical history included hypertension, chronic kidney disease, and osteoporosis. The patient had no fertility concerns during her reproductive years, with 5 uneventful pregnancies. She underwent a vaginal hysterectomy with ovarian preservation for uterine prolapse at age 63 years.

### Case 2

A 25-year-old woman was referred to the reproductive endocrinology clinic with significant clinical and biochemical androgen excess. Her medical history included juvenile dermatomyositis, dyslipidemia, hepatic steatosis, steroid-induced hyperglycemia, hypertension, and obstructive sleep apnea. A diagnosis of severe insulin resistance syndrome (SIRS) was made because of acquired partial lipodystrophy in the context of dermatomyositis, an association previously described in the literature [5].

Oligomenorrhoea was reported since menarche at age 13 years, followed by secondary amenorrhoea for 2 years before presentation. She reported significant hirsutism requiring local hair removal on alternate days, with poor response to spironolactone 50 mg daily. Clinically, there was partial lipodystrophy affecting the limbs with relative truncal sparing and evidence of severe insulin resistance with acanthosis nigricans at the nape of neck, axillae, and antecubital fossae.

### Case 3

A 67-year-old multiparous woman was referred with a 15-year history of hirsutism. This had previously been investigated in another institution without definitive pathology identified and required daily hair removal. She reported frontal hair thinning. Her medical history was significant for primary hypothyroidism and hypertension. There was no history of previous fertility concerns. Clinical examination showed an increased body mass index of 48.2 kg/m^2^, with no features of insulin resistance or Cushing syndrome.

## Diagnostic Assessment

### Case 1

Biochemical investigations showed significant serum testosterone (T) elevation at 41.3 nmol/L (1190.2 ng/dL; ref. 0.14-1.14 nmol/L); androstenedione (A4) and dehydroepiandrosterone sulfate levels were 9.43 nmol/L (270.2 ng/dL; 1.39-9.77 nmol/L) and 2.9 μmol/L (107.41 μg/dL; 0.3-6.7μmol/L), respectively. FSH and LH were 18.5 IU/L and 10.1 IU/L, respectively. Hematocrit was elevated at 0.504 L/L, with a hemoglobin of 16.4 g/dL (11.5-15 g/dL). An overnight dexamethasone suppression test was performed, with appropriate suppression of serum cortisol to less than 50 nmol/L (1.81μg/dL). Given the severe biochemical androgen excess, a prompt workup was initiated for localization. A transvaginal pelvic ultrasound showed a right ovarian hypoechoic vascular mass measuring 2.8 × 1.5 × 1.4 cm. A noncontrast computed tomography (CT) scan of the adrenals demonstrated normal symmetrical adrenal glands. In the context of imaging highlighting potential ovarian pathology, combined with preferential elevation of ovarian androgens (T >> A4), a GnRH suppression test was performed. A total of 3 mg of triptorelin was administered IM while awaiting gynecological input, with complete suppression of T and gonadotropins after 28 days (Table 1).

**Table 1.** Biochemical results of case 1 at presentation, after GnRH analogue suppression, and postoperatively following successful resection of benign ovarian steroid cell tumor

	Baseline pre-GnRH analogue	Day 28 post-GnRH analogue	Day 60 post-bilateral salpingo-oophorectomy
FSH	18.5 IU/L	2.8 IU/L	51.9 IU/L
LH	10.1 IU/L	<0.5 IU/L	26.6 IU/L
Testosterone	41.3 nmol/L (1190.2 ng/dL)	0.5 nmol/L (14.4 ng/dL)	<0.4 nmol/L (<11.5 ng/dL)
SHBG	58.2 nmol/L (6.5μg/mL)	75.7 nmol/L (8.5μg/mL)	82 nmol/L (9.2μg/mL)
DHEA-S	2.9μmol/L (107.4μg/dL)	1.5μmol/L (55.6μg/dL)	1.2μmol/L (44.4μg/dL)
Androstenedione	9.4 nmol/L (269.3 ng/dL)	0.2 (5.7 ng/dL)	1.75 (50.1 ng/dL)
Estradiol	169.0 pmol/L (46.0 pg/mL)	<92 pmol/L (25.06 pg/mL)	N/A

FSH, LH, and E2 reference (ref) ranges are dependent on phase of menstrual cycle and menopausal status. T ref: 0.14-1.14 nmol/L; SHBG ref: 27.1-128 nmol/L; DHEA-S ref: 0.3-6.7μmol/L; A4 ref: 1.39-9.77 nmol/L.

Abbreviations: N/A, not available; E2, estradiol.

### Case 2

Profound insulin resistance was confirmed with a fasting insulin level of 1208 pmol/L (173.9 μIU/mL) and fasting glucose of 5 mmol/L (90.1 mg/dL). Biochemical androgen excess was evident with serum T of 8.9 nmol/L (256.48 ng/dL) and A4 17.46 nmol/L (500.29 ng/dL) (Table 2). FSH and LH were 5.1 IU/L and 23.4 IU/L, respectively. Transabdominal sonographic imaging identified normal ovarian morphology. Noncontrast CT scan of the adrenals was unremarkable, and there was appropriate suppression of serum cortisol following an overnight dexamethasone suppression test. A GnRH analogue suppression test was therefore performed to confirm ovarian etiology. Following administration of triptorelin 3 mg, T was fully suppressed and A4 normalized (Table 2) after 28 days. The patient reported significant improvement in hirsutism during this timeframe.

**Table 2.** Biochemical results in case 2 with successful suppression of androgen excess following triptorelin 3 mg IM administration

	Baseline pre-GnRH analogue	Day 28 post-GnRH analogue
FSH	5.1 IU/L	6.3 IU/L
LH	23.4 IU/L	<0.1 IU/L
Testosterone	8.9 nmol/L (256.5 ng/dL)	<0.4 nmol/L (<11.5 ng/dL)
SHBG	60.1 nmol/L (6.8 μg/mL)	39.9 nmol/L (4.5 μg/mL)
DHEA-S	3.7 μmol/L (137.0 μg/dL)	3.7 μmol/L (137 μg/dL)
Androstenedione	17.5 nmol/L (501.4 ng/dL)	1.4 nmol/L (40.1 ng/dL)
Estradiol	331 pmol/L (90.2 pg/mL)	198 pmol/L (53.9 pg/mL)

FSH, LH, and E2 reference (ref) range are dependent on phase of menstrual cycle and menopausal status. T ref: 0.14-1.14 nmol/L; SHBG ref: 27.1-128 nmol/L; DHEA-S ref: 0.3-6.7μmol/L; A4 ref: 1.39-9.77 nmol/L.

Abbreviation: E2, estradiol.

### Case 3

Initial investigations confirmed biochemical androgen excess, with serum T 2.5 nmol/L (72.05 ng/dL), serum A4 of 5.94 nmol/L (170.2 ng/dL), and a dehydroepiandrosterone sulfate level of 2.5 μmol/L (92.59μg/dL) (Table 3). FSH and LH were 39.9 IU/L and LH 25.2 IU/L, respectively. A noncontrast CT scan of the adrenals was performed, with a 1.8-cm nodule identified, consistent with an incidental adrenal myelolipoma. Overnight dexamethasone suppression testing showed cortisol suppression below 50 nmol/L (1.81 μg/dL). Transvaginal ultrasound identified normal ovarian morphology. Given the symptom chronicity, combined with biochemical pattern of androgen excess, her presentation appeared consistent with ovarian disease, most likely ovarian hyperthecosis (OHT). GnRH suppression testing was performed, with 3 mg triptorelin administered intramuscularly. Twenty-eight days after administration, serum T was undetectable (Table 3).

**Table 3.** Therapeutic application of GnRH analogue suppression with successful androgen suppression in case 3

	Baseline pre-GnRH analogue	Day 28 post-GnRH analogue
FSH	39.9 IU/L	2.8 IU/L
LH	25.2 IU/L	1.3 IU/L
Testosterone	2.5 nmol/L (72.1 ng/dL)	1.1 nmol/L (31.7 ng/dL)
SHBG	35.0 nmol/L (3.9 µg/mL)	36.4 nmol/L (4.1 µg/mL)
DHEA-S	2.5 µmol/L (92.6 µg/dL)	2.1 µmol/L (77.8 µg/dL)
Androstenedione	5.9 nmol/L (170.2 ng/dL)	5.2 nmol/L (150.1 ng/dL)
Estradiol	130 pmol/L (35.4 pg/mL)	<92 pmol/L (<25.1 pg/mL)

Note preferential elevation in ovarian over adrenal androgens. FSH, LH, and E2 reference (ref) range are dependent on phase of menstrual cycle and menopausal status. T ref: 0.14-1.14 nmol/L; SHBG ref: 27.1-128 nmol/L; DHEA-S ref: 0.3-6.7 μmol/L; A4 ref: 1.39-9.77 nmol/L.

## Treatment

### Case 1

In light of the sonographic findings, coupled with results of the GnRH suppression test, this patient was referred to a specialist gynecologist and underwent a bilateral salpingo-oophorectomy. Suppression of T and gonadotrophins with GnRH analogue testing was reassuring and more indicative of benign pathology. Theoretically, failure of suppression would have incurred a more oncological surgical resection from concern regarding malignancy.

### Case 2

Given successful GnRH analogue-induced suppression of serum T and reduction in hirsutism, regular triptorelin 3 mg monthly was commenced, with add-back replacement with transdermal estrogen in tandem with cyclical oral micronized progesterone.

### Case 3

Initially, referral for curative bilateral oophorectomy was discussed. However, the patient elected to proceed with a therapeutic trial of GnRH analogue suppression, which was administered as triptorelin 3 mg once monthly.

## Outcome and Follow-up

### Case 1

Histology confirmed a 2-cm lipid-rich tumor, consistent with a benign ovarian steroid cell tumor, with ensuing resolution of hirsutism and suppression of postoperative serum T to <0.4 nmol/L (<11.5 ng/dL). Subjectively, voice deepening improved after 6 months.

### Case 2

In view of the clinical response, and no desire for future fertility, the patient has elected for a bilateral oophorectomy for sustained symptom control.

### Case 3

The patient was transitioned to long-acting depot preparation of triptorelin (11.25 mg every 3 months) following improvement in alopecia and reduction in frequency of hair removal.

## Discussion

The featured cases highlight the clinical application of GnRH analogues in diagnosing and managing of women with severe hyperandrogenism. Case 1 highlights a diagnostic role in which GnRH analogue administration was used to confirm gonadotropin-dependent ovarian androgen excess, with subsequent curative bilateral salpingo-oophorectomy. Refractory hyperandrogenism secondary to SIRS can be challenging to treat, and in case 2, we report significant improvement in symptoms within 1 month of treatment. This presents an option for fertility-sparing treatment in premenopausal cohorts. This contrasts with case 3, in which the patient experienced severe postmenopausal androgen excess. Despite a potential for curative surgery, our patient opted for a trial of medical therapy.

GnRH analogue testing assesses serum testosterone suppression following administration of a single dose of leuprorelin (3.75 mg), triptorelin (3 mg), or an equivalent subcutaneously or intramuscularly [3, 6]. Several cases in the literature have highlighted its diagnostic and therapeutic application in managing adverse effects in women [6, 7].

The principle underpinning this diagnostic test relies on ovarian hyperandrogenism remaining under regulatory control of the hypothalamic-pituitary-gonadal axis, despite supra-physiological androgen secretion. On administration, pituitary GnRH receptors undergo initial activation, with subsequent desensitization and downregulation. Gonadotropins, estradiol and T levels are monitored 21 to 28 days after administration. Suppression of T by more than 50% following GnRH analogue increases the likelihood of a benign source of ovarian hyperandrogenism [8]. Theoretically, GnRH testing should differentiate between OHT and virilizing ovarian tumor (VOT) from autonomous androgen secretion in the context of VOTs, with failure to suppress T after GnRH raising the suspicion of underlying malignancy [7, 8]. However real-world data highlight significant overlap in serum T response to GnRH analogues in patients with OHT and VOT [4].

The role for therapeutic use of GnRH analogues has been previously described in patients with SIRS [5]. In severe insulin resistance, profound stimulation of ovarian androgen production is driven by a synergistic association between LH and hyperinsulinemia, with preserved insulin sensitivity at the level of the ovary. The therapeutic mechanism of GnRH analogue efficacy uses the same physiological response of serum T observed diagnostically.

With advancing age, there is an increasing burden of adrenal “incidentalomas,” and pelvic imaging can often be inconclusive because of small lesion size. GnRH analogues are beneficial in postmenopausal women with a presentation consistent with benign ovarian hyperandrogenism, particularly those that present as poor surgical candidates because of excess operative morbidity, or patient preference [4, 6-10]. Similarly, they may be used temporarily in premenopausal women with refractory hyperandrogenism in the absence of lateralization, in which fertility preservation remains a priority. In this context, a “block and replace” strategy is used, with add-back hormone replacement to alleviate vasomotor symptoms and as a bone protective strategy [10]. However, this approach is not appropriate in premenopausal women with a suspected unilateral or potentially malignant source of ovarian androgen excess, who should be offered simultaneous ovarian and adrenal vein sampling for localization. Despite proving a beneficial adjunct in the workup of benign ovarian hyperandrogenism, screening for adrenal sources remains an important consideration, including a thorough assessment for cushingoid features, and adrenal imaging and dexamethasone suppression testing, as indicated. Currently, there is no clear delineation of the optimal use of GnRH analogue testing in current diagnostic algorithms for hyperandrogenism and is used predominantly in challenging cases. Given current lack of diagnostic cutoffs, caution should be used when interpreting results in the context of premenopausal women because of implications for fertility with definitive surgery. Results should be evaluated in the overall clinical context and in conjunction with additional investigations including imaging and pattern of androgen elevation.

## Learning Points

- GnRH analogue suppression testing is a useful adjunct in the diagnosis of suspected ovarian hyperandrogenism. Suppression of testosterone occurs because of preservation of gonadotropin regulation of ovarian androgen generation, even in the context of many virilizing ovarian tumors. Inclusion in diagnostic algorithms may reduce reliance on more invasive procedures, such as ovarian and adrenal vein sampling, in complex cases.
- Postmenopausal women with refractory symptoms of androgen excess secondary to benign ovarian hyperandrogenism may benefit from a trial of GnRH analogue therapy, particularly in those with high operative risk or reluctance to undergo surgery. They present a fertility-preserving option for premenopausal women with benign ovarian hyperandrogenism.
- Severe insulin resistance syndromes should be considered in premenopausal women with extreme ovarian androgen excess.
- Virilizing features should be sought in all cases of androgen excess to ensure malignancy is not overlooked.

## Data Availability

Data sharing is not applicable to this article as no datasets were generated or analyzed during the current study.

## Abbreviations

A4: androstenedione
CT: computed tomography
OHT: ovarian hyperthecosis
PCOS: polycystic ovary syndrome
SIRS: severe insulin resistance syndrome
T: testosterone
